# Extracting myricetin and dihydromyricetin simultaneously from *Hovenia acerba* seed by Ultrasound-Assisted extraction on a lab and small Pilot-Scale

**DOI:** 10.1016/j.ultsonch.2023.106304

**Published:** 2023-01-18

**Authors:** Xiaonan Zhang, Lubin Zhang, Yingdi Zhang, Tingting Xiong, Yaqian Niu, Yan Huang

**Affiliations:** a Jiaying University, Meizhou 514015, China; b Guangdong Provincial Key Laboratory of Conservation and Precision Utilization of Characteristic, Agricultural Resources in Mountainous Areas, Meizhou 514015, China; c Northeast Agricultural University, Food Science College, Harbin 150030, China

**Keywords:** *Hovenia acerba* seed, Ultrasonic-assisted extraction, Response surface analysis, Small pilot-scale, Myricetin and dihydromyricetin

## Abstract

The flavonoids myricetin and dihydromyricetin are significant components of *Hovenia acerba* seed. In this work, myricetin and dihydromyricetin were extracted from *Hovenia acerba* seed using an ultrasound-assisted technique, and the extraction parameters were adjusted using the response surface design approach. HPLC was used to assess the yield of myricetin and dihydromyricetin. According to the data, myricetin and dihydromyricetin yields were 0.53 mg/g and 4.06 mg/g at a 60 % ethanol solution concentration, 180 W of ultrasonic irradiation power, a 20 mL/g ratio of liquid to solid, and a 40 °C optimal extraction temperature. The aforementioned findings are virtually in agreement with the experimental findings suggested by the model. The study mentioned above thus offers a means of separating and developing useful components of natural goods.

## Highlights

• Myricetin and dihydromyricetin were simultaneously extracted by ultrasound assisted.
• Lab-scale ultrasound-assisted extraction is more efficient than small-pilot scale.
• Methods utilizing ultrasound are more effective than alternative approaches.

## 1 Introduction

Alcoholism, oxidative stress, and hepatic poisoning are now prevalent epidemics that will eventually progress to chronic conditions and shorten life spans [[1], [2]]. Alcohol toxicity and liver damage are the results of excessive drinking [[3], [4]]. After entering the body, ethanol and alcohol flow into the liver cells where they undergo an oxidation process and activate alcohol dehydrogenase, which produces acetaldehyde. When acetaldehyde is present in significant amounts, it can harm humans [5]. Hepatocyte steatosis is caused by continuous drinking, hepatocyte necrosis is caused by binge drinking [6], and cirrhosis is caused by chronic drinking [7].

**Fig. 1:**
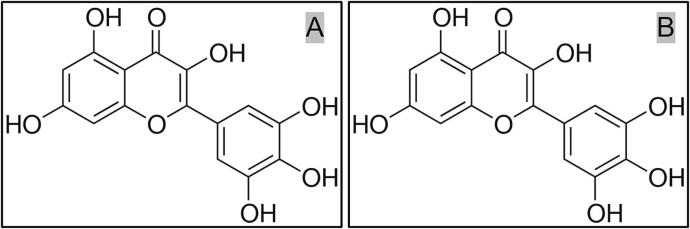
Chemical structures of myricetin (A) and dihydromyricetin (B).

*Hovenia acerba* (HA), a tall tree that belongs to the raisin family *Hovenia Thunb.*, is mostly found in China’s Yellow River and Yangtze River basins as well as South Korea and Japan. The *Hovenia acerba* seed (HAS) is frequently used in therapeutic settings as a treatment for symptoms of alcohol poisoning, including nausea, dizziness, exhaustion, and polydipsia [[8], [9]]. Myricetin and dihydromyricetin (see Fig. 1) are flavonoids [10], which have been shown to treat alcohol poisoning, prevent alcoholic liver, block liver cell deterioration, lower the risk of liver cancer, and lower three high levels [11].

Traditional plant extraction methods include microwave-assisted extraction [12], air flow crushing extraction [13], Soxhlet extraction [14], etc. However, irradiating raw materials for an extended period of time with microwaves requires a lot of energy, and focused irradiation produces too much heat, which denaturizes flavonoids and other heat-sensitive chemicals. The target product is extremely easily broken down, which reduces the effectiveness of the extraction. Long-term storage often takes place in a dry atmosphere, similar to the raw materials for extraction. A lot of powder and dust will be produced during the mechanical crushing process used to extract plants, which will damage human respiratory systems and pollute the environment [15].

The ecologically friendly ultrasonic assisted extraction approach boosts extraction stability while simultaneously speeding up extraction rates. In the ultrasonic process, the extraction medium will produce a lot of bubbles. According to Li et al. and Shirsath et al. [[16], [17]], when a cavitation bubble ruptures close to a cell wall, the explosive force creates a liquid jet phenomenon and the pressure created upon contact with the cell wall causes the cell to rupture, allowing the solvent to effectively enter the cell interior [[18], [19]]. Due to its quick extraction time, great efficiency, and attention to environmental preservation, ultrasonic technology is regarded as a “green” extraction method [20] The thermally unstable material and low temperature extraction industries make extensive use of ultrasound-assisted extraction technologies. With further study, ultrasound-assisted surgery will become more common in the sectors of food, medicine, chemical manufacturing, and other industries [21]. Additionally, ultrasonography is a green technology that is frequently utilized to replace gas emissions and prevent the greenhouse impact, making it a crucial future growth path.

Additionally, the heat produced by the high-speed comminution process will have an impact on the thermally sensitive material. In recent years, there has been a lot of interest in using ultrasonic technology as a green extraction technique to separate natural compounds from plant materials [[22], [23], [24], [25]]. With its energy-saving, high-efficiency, and robust operability, ultrasonic technology will cause the cavitation phenomenon, which will result in the formation of millions of tiny bubbles in the liquid, their growth, and eventual rupture. During this process, the material is affected by the cavitation phenomenon, which results in the building up of pressure inside the bubble and bubble burst, which causes the expansion and rupture of plant cell walls to serve the intended purpose [[26], [27]]. To extract myricetin and dihydromyricetin simultaneously using multiple parameters (ultrasonic temperature, duration, power/liquid ratio, etc.), we attempted using an ultrasonic-assisted approach in accordance with the study mentioned above. The majority of organic solvents are expensive, leave solvent residues in the active substances, and pollute the environment during usage. As a result, choosing the right extraction solvents is essential for maintaining the biological activity of extracts [28]. The ethanol solution was chosen as the extraction solvent because it is a great environmentally friendly and green solvent and because it can be recovered after extraction. Ethanol is a suitable choice as a modifier because it is non-toxic, food-grade, and in addition, the ethanol solution was chosen because it is a great environmentally friendly and green solvent. This research approach will be extremely helpful for the progress of extraction technology in other industries in the future. Furthermore, due to the relatively poor extraction efficiency and stability of myricetin and dihydromyricetin, ethanol solutions are typically favored over aqueous solutions [29].

It has not before been reported in the literature that small pilot-scale investigations on the direct extraction of myricetin and dihydromyricetin from HAS have been conducted. Pingret et al. (2012) demonstrated that using ultrasonic assisted extraction (20 kHz, 40 °C, 40 min) enhanced polyphenol extraction rates from apple slag in a 30 L tank by 30 % compared to using traditional extraction [30]. Here, we compared how ultrasound affected the production of myricetin and dihydromyricetin in HAS. We predicted that, as had been shown on a lab-scale, improvements in extraction yield would also be seen on a small-pilot scale.

## 2 Materials and methods

### 2.1 Materials

From Johnson Chemical Co., Ltd, chromatographic pure methanol was bought (Beijing, China). Purchased from ANPEL Laboratory Technologies lnc are myricetin and dihydromyricetin (purity 99 %, HPLC) (Shanghai, China). Other chemicals were analytical-grade and were purchased from Beijing Chemical Reagents (Beijing, China). Sankeshu Medical Materials Market was the source of the acquisition for HAS (Harbin, China).

### 2.2 Pretreatment of raw materials

The HAS were cleaned of mechanical impurities (sand, grass, sticks, etc.), crushed, and screened through a 40 mesh screen to create a homogenous powder for usage. 100 g of raw material underwent 1000 mL of petroleum ether, which was then filtered after being ultrasonically processed for an hour. For reserve usage, the degreasing raw material was let to dry for 24 h on a flat plate.

### 2.3 Ultrasound-assisted extraction on lab-scale

First, ethanol solution was used to disperse HAS powder (feeding range: 0.333–1 g), with volume and concentration being the variables. The ultrasonic extraction process took place for 30 min and an operating frequency of 45 kHz, a power range of 32–180 W and a temperature range of 30–60 °C. After extraction was finished, the extraction fluid was centrifuged for 8 min at 5000 rpm after being naturally cooled to 25 °C. In order to measure the content of myricetin and dihydromyricetin in the samples using HPLC, the supernatant was collected and filtered for 0.22 μm.

### 2.4 Response surface model building and statistical analysis

The solid–liquid ratio (A), ultrasonic irradiation power (B), and ultrasonic irradiation time (C) were three parameters with considerable single factor effect that were optimized using the response surface Box-Behnken (Expert 11.0) approach. The experiment was planned using a response surface analysis with three factors and three levels, as indicated in Table 1.

**Table 1:** Factors and levels design of Box-Behnken design.

Code	Factors	Levels		
		−1	0	1
X_1_	Liquid to solid ratio (mL/g)	15	20	25
X_2_	Ultrasonic irradiation power (W)	108	144	180
X_3_	Ultrasonic irradiation time (min)	20	30	40

### 2.5 Ultrasound-assisted extraction on a small-pilot scale

Utilizing the CXN-2C ultrasonic extractor, a small-pilot scale ultrasonic extraction experiment was conducted (Beijing, China). The ultrasonic equipment had a total volume of 2.5 L, an effective working volume of 2.1 L, an ultrasonic frequency of 40 kHz, a continuously adjustable ultrasonic irradiation power, and a maximum ultrasonic irradiation power of 1200 W (Extraction at room temperature, and ultrasonic irradiation times were 1, 3, and 5 h). On the control panel, it is possible to change the reaction system temperature, stirring speed, ultrasonic irradiation power, and ultrasonic irradiation time. Each batch contained 50 g of HAS powder, which was mixed in a small pilot scale ultrasonic auxiliary device (compared to the last laboratory investigation, the amount of raw material was multiplied by 50). In order to identify myricetin and dihydromyricetin by HPLC, the extract was centrifuged at 5000 rpm for 15 min. The supernatant was then filtered over a 0.22 μm membrane.

### 2.6 Determination of myricetin and dihydromyricetin by HPLC.

The standard curve method was used to determine the contents of myricetin and dihydromyricetin [31]. HPLC chromatographic analysis was performed for reference after 2 mL of supernatant was obtained and filtered through 0.45 μm. The chromatographic column was C-18, 4.6 mm × 250 mm × 5 μm, Kya technology, and the mobile phase was methanol-0.02 moL/L potassium dihydrogen phosphate. The temperature was 25℃, and myricetin and dihydromyricetin were detected.

## 3 Results and discussion

### 3.1 Single factor extraction process

**Fig. 2:**
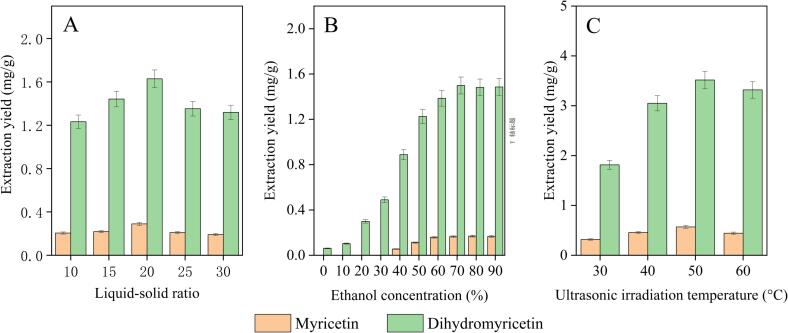
Effect of various variables on the yield of myricetin and dihydromyricetin extraction.

This portion of the investigation was conducted under the following conditions: ultrasonic duration was 30 min; ultrasonic temperature was 30 °C; ultrasonic irradiation power was 180 W; liquid to solid ratio was 20 mL/g; and solvent dose was 10 mL. The production of myricetin and dihydromyricetin peaked at a ratio of 20 mL/g, as shown in Fig. 2A, and then steadily declined as the ratio increased. A big enough amount of solvent might partially dissolve the flavonoid molecules, which would be detrimental to the extraction procedure. As a result, the 20 mL/g liquid–solid ratio was our next research of choice.

The amount of organic solvent used in the extraction experiment is crucial. We looked at how different gradients of ethanol concentration (0–90 %) affected the yield. Myricetin and dihydromyricetin had the maximum extraction yields at a concentration of 50 % ethanol (Fig. 2B), but as the concentration rose, flavonoids were more readily dissolved in ethanol [32]. There were few additional changes at the alcohol concentration of 80 % as the concentration increased because the improvement in the extraction rate slowed down, perhaps because the majority of the raisin components had already been drained [33]. Fig. 2C shows that the relationship between the extraction rate and temperature of the ultrasonic extraction was nearly entirely positive. The production of myricetin and dihydromyricetin grew gradually as the temperature rose, but at 60 °C, the yield of the two substances fell. Under high temperature circumstances, some myricetin and dihydromyricetin may cause degradation or isomerization. As a result, we decided to conduct the follow-up experiment at a temperature of 40 °C.

**Fig. 3:**
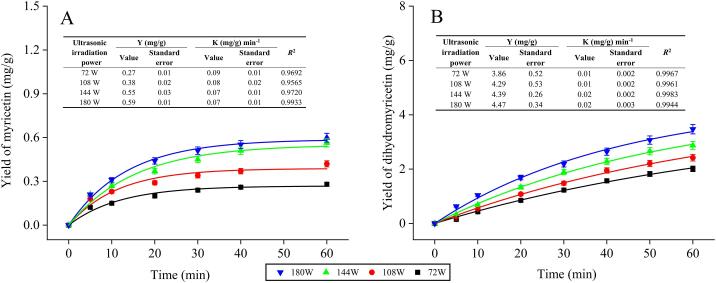
Extraction of kinetic curve: (A) myricetin and (B) dihydromyricetin.

A first-order and second-order fitting dynamic curve model is used in the ultrasound-assisted extraction of dynamic data (Fig. 3). The kinetic curves for myricetin and dihydromyricetin in the two figures are obviously practically identical. Myricetin content was rather low, as evidenced by the fact that the yield of the myricetin extraction grew significantly at first and then stabilized after 30 min of ultrasonic irradiation (Fig. 3A). After 60 min, the production of dihydromyricetin was 3.47 mg/g. The aforementioned findings demonstrated that myricetin was significantly affected by the ultrasonic-assisted extraction process. Most of the components in the sample were released when the ultrasonic irradiation time was 30 min, yielding 0.51 mg/g of myricetin. Myricetin yield remained virtually unchanged throughout the course of 60 min at 0.56 mg/g. Due to the low myricetin level, the yield of the brief extraction remained unchanged. However, when the ultrasonic irradiation period was 60 min, the dihydromyricetin extraction rate release was slow and the amplitude was modest (Fig. 3B). It required more time and power to finish since the yield of dihydromyricetin was positively connected with time and power and the growth pattern was sluggish and consistent. It was clear that the breakdown of functional components in cells was hastened by the immediate pressure brought on by ultrasonic cavitation, which also caused the cell wall to rupture and the cell body to disintegrate [34].

### 3.2 Parameter optimization by response surface methodology

Response surface technique BBD was used to optimize the extraction parameters based on a single factor experiment in order to achieve the optimal ultrasonic-assisted extraction conditions, and the subsequent impacts and interactions of these variables on the yield were investigated [35].

Table 2 and Table 3 display the results of the 17 experiments using the extraction parameters liquid–solid ratio, ultrasonic irradiation time, and ultrasonic irradiation power. The tables display the experimental data as well as the outcomes of the predictions. We use response surface analysis to fit the data in Table 2 and Table 3 were obtain the regression equation:

(1) $$Y=0.52+0.01X_{1}+0.03X_{2}-0.02X_{3}-0.09X_{1}X_{2}-0.06X_{1}X_{3}-0.07X_{2}X_{3}-0.13X_{1}^{2}-0.12X_{2}^{2}-0.14X_{3}^{2}$$

(2) $$Y=4.06+0.12X_{1}+0.07X_{2}-0.03X_{3}-0.06X_{1}X_{2}-0.70X_{1}X_{3}-0.71X_{2}X_{3}-1.02X_{1}^{2}-1.06X_{2}^{2}-1.06X_{3}^{2}$$

**Table 2:** Box-Behnken design with experimental value for myricetin, analysis of varance (ANOVA) for reponse surface quadratic model.

Run	BBD experiments				ANOVA								
	X_1_	X_2_ (w)	X_3_ (min)	Y_1_ (mg/g)	Source of variation	Sum of squares	Degree of freedom	Mean square	F-value	P-value			
1	15	144	20	0.19	**Model**	0.32	9	0.04	87.77	< 0.0001**			
2	15	108	30	0.16	**X_1_**	2.2 × 10^-4^	1	2.2 × 10^-4^	0.55	0.4821			
3	20	144	30	0.55	**X_2_**	9.2 × 10^-3^	1	9.2 × 10^-3^	22.93	0.0020			
4	20	180	20	0.40	**X_3_**	2.1 × 10^-3^	1	2.1 × 10^-3^	5.20	0.0567			
5	25	144	40	0.21	**X_1_^2^**	0.07	1	0.07	185.09	< 0.0001**			
6	20	180	40	0.21	**X_2_^2^**	0.06	1	0.06	155.02	< 0.0001**			
7	25	108	30	0.32	**X_3_^2^**	0.08	1	0.08	205.87	< 0.0001**			
8	20	144	30	0.52	**X_1_X_2_**	0.03	1	0.03	72.62	< 0.0001**			
9	20	144	30	0.52	**X_1_X_3_**	0.01	1	0.01	33.91	0.0006			
10	25	180	30	0.22	**X_2_X_3_**	0.02	1	0.02	44.86	0.0003			
11	15	180	30	0.40	**Residual**	2.8 × 10^-3^	7	4.0 × 10^-4^					
12	15	144	40	0.30	**Lack of fit**	1.9 × 10^-3^	3	6.4 × 10^-4^	2.95	0.1618			
13	20	144	30	0.52	**Pure error**	8.7 × 10^-4^	4	2.2 × 10^-4^					
14	20	108	20	0.20	**Corrected total**	0.32	16						
15	25	144	20	0.34	**Credibility analysis of the regression equations**								
16	20	108	40	0.28	**Index mark**	Standard deviation	Mean	CV%	Press	R^2^	Adjust R^2^	Predicted R^2^	Adequacy precision
17	20	144	30	0.54	**Y**	0.02	0.35	5.79	0.032	0.9912	0.9799	0.8990	24.66

X1: Liquid-solid ratio (mL/g); X2: Ultrasonic irradiation power (w); X3: Ultrasonic irradiation time (min); Y1: Yield of myricetin. “**”: Extremely Significant.

**Table 3:** Box-Behnken design with experimental value for dihydromyricetin, analysis of varance (ANOVA) for reponse surface quadratic model.

Run	BBD experiments				ANOVA								
	X_1_	X_2_ (w)	X_3_ (min)	Y_2_ (mg/g)	Source of variation	Sum of squares	Degree of freedom	Mean square	F-value	P-value			
1	15	144	20	1.26	**Model**	21.34	9	2.37	123.62	< 0.0001**			
2	15	108	30	1.33	**X_1_**	0.12	1	0.12	6.02	0.0439			
3	20	144	30	4.13	**X_2_**	0.04	1	0.04	2.17	0.1843			
4	20	180	20	2.69	**X_3_**	6.3 × 10^-3^	1	6.3 × 10^-3^	0.33	0.5853			
5	25	144	40	1.30	**X_1_^2^**	4.37	1	4.37	227.92	< 0.0001**			
6	20	180	40	1.25	**X_2_^2^**	4.74	1	4.74	247.09	< 0.0001**			
7	25	108	30	2.99	**X_3_^2^**	4.77	1	4.77	248.84	< 0.0001**			
8	20	144	30	3.99	**X_1_X_2_**	1.72	1	1.72	89.73	< 0.0001**			
9	20	144	30	4.12	**X_1_X_3_**	1.94	1	1.94	101.37	< 0.0001**			
10	25	180	30	1.33	**X_2_X_3_**	2.00	1	2.00	104.01	< 0.0001**			
11	15	180	30	2.29	**Residual**	0.13	7	0.02					
12	15	144	40	2.57	**Lack of fit**	0.11	3	0.04	6.09	0.057			
13	20	144	30	4.10	**Pure error**	0.03	4	6.0 × 10^-3^					
14	20	108	20	1.21	**Corrected total**	21.48	16						
15	25	144	20	2.78	**Credibility analysis of the regression equations**								
16	20	108	40	2.60	**Index mark**	Standard deviation	Mean	CV%	Press	R^2^	Adjust R^2^	Predicted R^2^	Adequacy precision
17	20	144	30	3.99	**Y**	0.14	2.58	5.37	1.80	0.9937	0.9857	0.9162	27.60

^X1: Liquid-solid ratio (mL/g); X2: Ultrasonic irradiation power (w); X3: Ultrasonic irradiation time (min); Y2: Yield of dihydromyricetin. “**”: Extremely Significant.^

In the extraction of myricetin and dihydromyricetin (P＜0.0001), the impacts of secondary terms and interaction terms were very significant. The impacts of each extraction condition on the extraction effect of dihydromyricetin were successively as follows, as determined by comparing the F value in the major item of Table 2: liquid–solid ratio (X_1_), ultrasonic irradiation power (X_2_), and ultrasonic irradiation time (X_3_). While the other key components did not have a substantial impact on extraction, the liquid–solid ratio did. Similar to this, myricetin’s impacts on ultrasonic irradiation power are sequentially X_2_, X_3_, and X_1_, and these effects pass the significance test. The response Y (dihydromyricetin and myricetin yields of second-order polynomial equations have low P values (＜0.0001) and F values (123.62 and 87.77), and Lack of fit was not significant (P > 0.05). These results demonstrate the ideality of the second-order polynomial equation and the significance of the model height [36]. The obtained second-order polynomial regression model can fully explain the steps of this experiment, as can be seen from the table, where R^2^ (0.9937, 0.9912) shows that the predicted value and the experimental value fit well with high correlation and the adjust R^2^ and predicted R^2^ are high and similar. Furthermore, the C.V. is 5.37 %. The fitted regression equation conforms with the test principle and has high adaptability, as evidenced by the precise precision of 27.60 [35]. Through the fitting equation, we may further extract the outcomes of the experimental optimization.

**Fig. 4:**
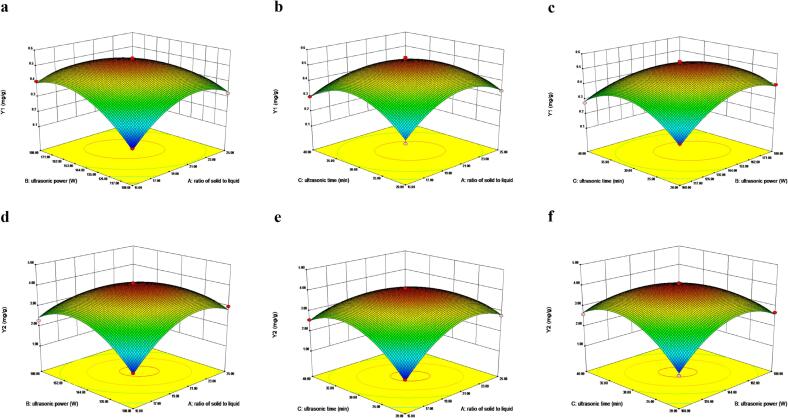
Three-dimensional figures of interactive effect on yield of myricetin (a: ultrasonic irradiation power and liquid–solid ratio, b: ultrasonic irradiation time and liquid–solid ratio, c: ultrasonic irradiation time and ultrasonic irradiation power;) and dihydromyricetin (d: ultrasonic irradiation power and liquid–solid ratio, e: ultrasonic irradiation time and liquid–solid ratio, f: ultrasonic irradiation time and ultrasonic irradiation power).

Fig. 4a, b, and c, as well as Fig. 4d, e, and f, are three-dimensional depictions of the interactive effect on the yield of myricetin and dihydromyricetin, respectively. As ultrasonic irradiation time and power increased, the yield of myricetin and dihydromyricetin also displayed a trend of increasing at first and then gradually declining (Fig. 4b and c). When the liquid–solid ratio and power grow, they do so plainly initially before decreasing (Fig. 4c and d). The interplay of liquid–solid ratio and extraction duration on myricetin and dihydromyricetin extraction rates are depicted in Fig. 4e and f. The extraction yield rose quickly and gradually with an increase in the liquid–solid ratio and ultrasonic irradiation time. Additionally, the figure’s structure highlights the importance of the interactions between the three extraction factors. The extraction rate significantly increased, as shown in Fig. 4d and e, and the liquid–solid ratio had a substantial impact on the extraction. As a result, the interaction between ultrasonic irradiation power, ultrasonic irradiation time, and liquid–solid ratio was considerable and contributed to the experiment’s accuracy.

The following were the ideal theoretical outcomes produced by BBD response surface optimization: The liquid–solid ratio was 20.20 mL/g, the ultrasound irradiation time was 29.40 min, and the ultrasonic irradiation power was 146 W. Dihydromyricetin yielded 4.06 mg/g under these circumstances, whereas myricetin yielded 0.53 mg/g. We carried out the verification experiment using a liquid–solid ratio of 20 mL/g, an ultrasonic power of 146 W, and an ultrasonic duration of 29 min. The yields of myricetin and dihydromyricetin were 4.04 mg/g and 0.05 mg/g, respectively, which were about in line with what BBD expected. Therefore, it was anticipated that the aforementioned strategy would work.

### 3.3 Recovery of solvent

Ethanol solution, the organic solvent employed in this experiment, is split into two parts in the residual material, one of which is the cleared solution following filtering extraction of the solute and the other of which is the solvent in the residue. To remove the solvent, the two portions were mixed first, and then the rotary evaporation was done at 60 °C with a vacuum of 0.08 MPa (Shanghai Shensheng R002B, China). The remaining concentrated solution was cooled to ambient temperature, centrifuged at 3000 rpm, and the supernatant was collected. The remaining concentrated solution was then dried for 24 h at 60 °C. The experiment was run three times, and by calculating the average recovery of the three runs, the ethanol’s recovery rate, the recovered ethanol was frequently utilized [37].

### 3.4 Comparison with the traditional methods

The primary determinant in this investigation was the efficiency at which myricetin and dihydromyricetin were extracted [38]. The Ionic liquid homagenate extraction, the microwave-assisted ethanol extraction, and the heated reflux extraction were contrasted with the ultrasonic aided extraction technique, in this process, ethanol is used as the extraction solvent.

As is shown in the Table 4, the features of the heating extraction approach include a lengthy extraction period and a decreased yield of myricetin and dihydromyricetin, which may be because heat-sensitive molecules are broken down during this procedure; However, the homogenate approach and ultrasonic-assisted method are appropriate for substances that are thermally unstable. In comparison to homogenate extraction, ultrasound-assisted extraction had a higher solvent recovery, produced a relatively high yield in a short amount of time. Target chemicals vibrated more quickly in the extraction solvent, which reduced the ultrasonic irradiation time and produced superior extraction outcomes. Extractive solvents outside the mass transfer unit contents induce cavitation phenomena here, cell rupture and heat transfer create severe thermal stress and local high pressure, and conduction and convective heat transfer here are what determine the yield of myricetin and dihydromyricetin.

**Table 4:** Comparing several extraction techniques using ethanol as the solvent.

Method	Concentration of ethanol (%)	Temperature (℃)	Energy saving	Yield (mean ± SD, mg/g)	
				Myricetin	Dihydromyricetin
Ultrasound assisted extraction	65 %	Room temperature	Yes	0.51 ± 0.08	4.04 ± 0.05
Ionic liquid homagenate extraction	45 %	Room temperature	Yes	0.52 ± 0.16	4.69 ± 0.03
Microwave-assisted extraction	70 %	95	No	0.23 ± 0.18	3.58 ± 0.08
Heated reflux extraction	85 %	85	No	0.42 ± 0.15	0.79 ± 0.03

In light of the aforementioned considerations, the environmentally friendly, energy-saving, and environmental protection ultrasound-assisted extraction method avoids the dust pollution caused by the crushing process during processing. ultrasound-assisted myricetin and dihydromyricetin extraction offers a potent and effective alternative to conventional methods for the extraction of active substances from plants.

### 3.5 Comparison with the small-pilot scale

According to experiments, cavitation in liquid decreases in quantity and intensity as frequency increases, and greater amplitude values are achieved at lower frequencies with constant intensity. Accordingly, cavitation at 40 kHz was superior to that at 45 kHz in the small-pilot scale research because of the larger amplitude, thus higher yields of myricetin and dihydromyricetin should be achieved. However, in the small pilot scale test, the particle size of the raisin powder was appropriately enlarged to replicate the realistic operation in the big raisin process. As a consequence, a small portion of the solvent may have been overlooked in the test, which might have decreased the extraction rate. On pilot equipment, three 50 g amplification validation tests were conducted. Myricetin and dihydromyricetin had real yields of 0.38 ± 0.04 mg/g and 2.65 ± 0.11 mg/g, respectively. When comparing small pilot scale extraction to laboratory scale, the yield of myricetin and dihydromyricetin was much lower. This might be because the two systems differ in terms of particle size, operation frequency, shape, and mixing strength.

## 4 Conclusion

In this work, myricetin and dihydromyricetin were simultaneously extracted using the ultrasonic assisted technique. The single factor combined response surface approach was used to optimize the key variables, such as extraction concentration, liquid–solid ratio, reaction temperature, ultrasonic radiation power, and ultrasonic irradiation time, that impact the yield of the target analytical products. The experimental process’s consistency, repeatability, and solvent recovery were confirmed. We also contrasted the effectiveness of ultrasonic extraction on a small-pilot scale size vs a lab-scale. The ideal conditions for each experiment were as follows: 40 % extraction temperature, 60 % ethanol concentration, 40 min of ultrasonic irradiation time, 144 W of ultrasonic radiation power, and a 20 mL/g liquid to solid ratio. These conditions resulted in the highest yields of 4.06 mg/g and 0.53 mg/g of dihydromycetin, respectively. Myricetin and dihydromyricetin may be extracted simultaneously using ultrasound assistance, which is more reliable and stable than traditional extraction techniques. The lab-scale ultrasonic extraction method also has a greater extraction rate as compared to the small-pilot scale ultrasonic extraction method. This study offers a theoretical foundation for real manufacturing and use, making it appropriate for popularization.

## Declaration of Competing Interest

The authors declare that they have no known competing financial interests or personal relationships that could have appeared to influence the work reported in this paper.
